# Point-of-Care Ultrasound for Mimicker Lesions of Incarcerated Inguinal Hernia

**DOI:** 10.1155/2023/5593369

**Published:** 2023-09-06

**Authors:** Takahiro Hosokawa, Shinsuke Yoshizawa, Kyoichi Deie, Kensuke Ohashi, Hiroshi Kawashima

**Affiliations:** ^1^Department of Radiology, Saitama Children's Medical Center, Saitama, Japan; ^2^Department of Urology, Saitama Children's Medical Center, Saitama, Japan; ^3^Department of Surgery, Saitama Children's Medical Center, Saitama, Japan

## Abstract

Inguinal hernia is the most common surgical disease in pediatric patients, and urgent intervention such as manual reduction is needed for incarcerated inguinal hernia. Torsion of undescended testes, inguinal herniated ovarian torsion, and purulent lymphadenitis are mimickers of this condition. If these mimicker lesions are misdiagnosed as incarcerated inguinal hernia, manual reduction usually fails, and edematous and erythematous changes may occur in these mimicker lesions due to manual reduction. For physicians in the emergency department, prompt decisions and familiarity with the sonographic appearance of different contents within an inguinal hernia are important to accurately diagnose these mimickers. In this case series, we present sonographic images of a typical case of incarcerated inguinal hernia (an 11-month-old male with right incarcerated inguinal hernia) and three cases of mimicker lesions (a 7-month-old female with herniated ovarian torsion, a 7-year-old boy with undescended testicular torsion, and a 2-month-old male with purulent lymphadenitis). The incidence of incarcerated inguinal hernia is reported to be higher in males (80%), on the right side (60%), and in infants and toddlers. This information is important for diagnosing mimicker lesions. In addition, to prevent manual reduction in mimicker diseases, point-of-care ultrasound before manual reduction in suspected cases of incarcerated inguinal hernia is important.

## 1. Introduction

Inguinal hernia is the most common surgical disease in pediatric patients, and urgent intervention is needed for incarcerated inguinal hernia [1, 2]. The clinical symptoms of incarcerated inguinal hernias are irreducible, nonfluctuant bulges that are tender [1, 3]. Torsion of undescended testes, inguinal herniated ovarian torsion, and purulent lymphadenitis are mimickers of this condition [4, 5]. After diagnosis of an incarcerated inguinal hernia, urgent manual reduction is usually attempted in the emergency department [1]. However, if they are mimicker conditions misdiagnosed as incarcerated inguinal hernia, manual reduction usually fails, and edematous and erythematous changes may occur in these mimicker lesions due to manual reduction. Point-of-care ultrasound is useful in differentiating these mimicker lesions. For physicians in the emergency department, prompt decisions and familiarity with the sonographic appearance of different contents within an inguinal hernia are important to accurately diagnose these mimickers. In this case series, we present sonographic images of a typical case of incarcerated inguinal hernia and three cases of mimicker lesions that were challenging because manual reduction had been attempted.

## 2. Case Presentations

### 2.1. Case 1

**Figure 1:**
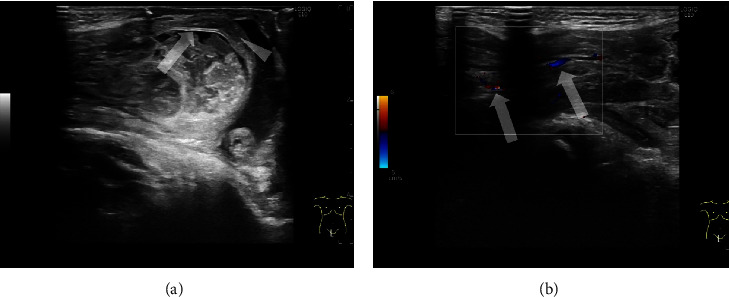
Case 1: an 11-month-old male with a right incarcerated inguinal hernia. (a) Sonogram of the right inguinal bulge showing a cystic structure accompanying intestinal wall layers. A high-echoic layer as mucosa (arrow) and a low-echoic layer (arrowhead) as submucosa or muscular layer can be visualized. Due to edematous changes in the intestinal wall, the five-layer structure of the intestinal wall could not be clearly visualized. (b) Color Doppler sonogram of the inguinal hernia shows vascular flow within the intestine (arrows). Intestinal peristalsis was preserved but weak.

An 11-month-old male with a right incarcerated inguinal hernia. He presented with a right inguinal bulge, vomiting, and crying. He was diagnosed with an incarcerated inguinal hernia (Figure 1). Manual reduction was successful.

### 2.2. Case 2

**Figure 2:**
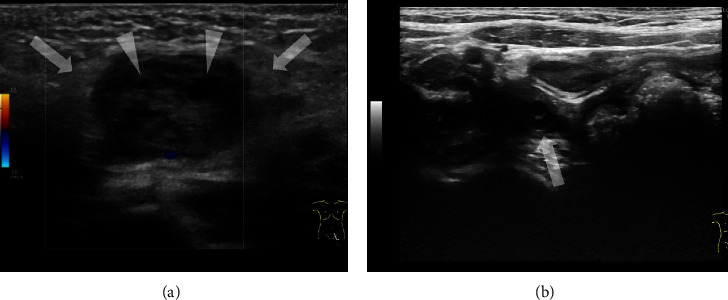
Case 2: a 7-month-old female with herniated ovarian torsion. (a) Sonogram of the left inguinal bulge showing a low-echoic mass with some cystic follicles (arrowheads). Color Doppler imaging shows diminished vascular flow within the mass. The surrounding subcutaneous tissue exhibits edematous changes (arrow). Therefore, herniated ovarian torsion was diagnosed and confirmed surgically. (b) Sonogram of the right ovary. Normal right ovary is detected in the pelvis. A few small cysts from ovarian follicles are evident (arrow).

A 7-month-old female with herniated ovarian torsion. She presented with a left inguinal bulge and inconsolable crying. She was diagnosed with an incarcerated inguinal hernia. Manual reduction was attempted but failed. Subsequently, ultrasound was performed, which showed multiple low-echoic cystic lesions with surrounding edematous subcutaneous tissue and diminished vascular perfusion (Figure 2). Therefore, herniated ovarian torsion was diagnosed and confirmed surgically.

### 2.3. Case 3

**Figure 3:**
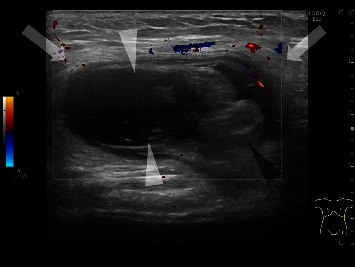
Case 3: a 7-year-old boy with undescended testicular torsion. Sonogram of the left inguinal bulge shows a low-echoic testis (white arrowheads) accompanied by swelling of the epididymis (black arrowhead). This low-echoic structure was not connected to the abdomen and was not detected to be a layer of the intestinal wall. Vascular perfusion within the testis and epididymis is diminished. The surrounding subcutaneous tissue exhibits edematous changes (arrows).

A 7-year-old boy with undescended testicular torsion. He had cerebral palsy due to premature birth. He presented with a left inguinal bulge and inconsolable crying. He was diagnosed with an incarcerated inguinal hernia. Manual reduction was attempted but failed. Subsequently, ultrasound was performed, which revealed a low-echoic testis accompanied by surrounding subcutaneous tissue edema. Vascular perfusion within the testis was diminished, and parenchymal echogenicity of the affected testis was low (Figure 3). Therefore, undescended testicular torsion was diagnosed and confirmed surgically.

### 2.4. Case 4

**Figure 4:**
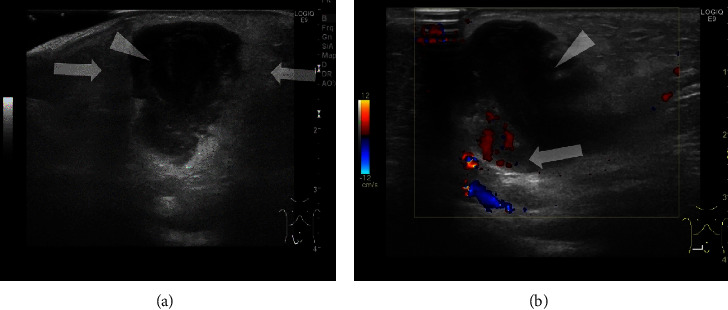
Case 4: a 2-month-old male with purulent lymphadenitis. (a) Sonogram of the right inguinal bulge shows a low-echoic lymph node (arrowhead) with surrounding effusion and high-echoic surrounding subcutaneous tissue (arrows). Therefore, purulent lymphadenitis was diagnosed. (b) Color Doppler sonogram at the inguinal lymph node shows the absence of vascular flow within the lesion (arrowhead). An adjacent enlarged lymph node is visualized (arrow).

A 2-month-old male with purulent lymphadenitis. He had a right inguinal bulge and was crying inconsolably. He was diagnosed with an incarcerated inguinal hernia. Manual reduction was attempted but failed. Subsequently, ultrasound was performed, which revealed a low-echoic lymph node with surrounding effusion and high-echoic surrounding subcutaneous tissue (Figure 4). Therefore, purulent lymphadenitis was diagnosed. A surgical incision and drainage were performed, and purulent lymphadenitis due to methicillin-resistant *Staphylococcus aureus* was confirmed.

## 3. Discussion

The incidence of incarcerated inguinal hernia is reported to be higher in males (80%), on the right side (60%), and in infants and toddlers (age 0-1 years (one-seventh), 1-2 years (one-seventh), and 2–5 years (one-third)) [1] (case 1). This information is important for diagnosing mimicker lesions. In addition, the presence of intestine as hernia content is a critical clue for diagnosis; therefore, in a sonogram, it is important to visualize the intestinal layer as intestinal wall structure and the connection of the intestine between the located inguinal hernia and intraabdominal cavity [6, 7]. In case 2, the patient was a “female” and had a “left side” lesion. Therefore, this was not a typical presentation of an incarcerated inguinal hernia [8]. In case 3, the patient had a history of cerebral palsy and was not a toddler. Approximately 60% of undescended testicular torsion is associated with cerebral palsy or neuromuscular diseases, and the patient's age was also not typical for incarcerated inguinal hernias [5, 9]. In case 4, it was difficult to differentiate purulent lymphadenitis from inguinal hernia incarceration. Inguinal lymphadenitis occurs at 1–4 years of age and has an age distribution similar to that of incarcerated inguinal hernias [1, 4]. In addition, the symptoms of inguinal lymphadenitis and incarcerated inguinal hernia are similar. A history of omphalitis or umbilical procedures is reported to be associated with the incidence of inguinal lymphadenitis (50%) [4]; however, the current case did not have this history.

Proper medical history and physical examination are important for the diagnosis of mimicker lesions, and point-of-care ultrasound can provide additional clues to help diagnose these lesions. In cases of torsion of an ovarian hernia, the inguinal lesion generally presents with multiple small cysts due to ovarian follicles, and Doppler imaging shows the absence of blood flow within the lesion [10]. Elective repair is recommended for ovarian herniation not accompanied by torsion or strangulation, whereas urgent repair is recommended for ovarian herniation accompanied by torsion or strangulation [11]. In cases of torsion of the undescended testis, ultrasound shows diminished blood flow within the undescended testis and abnormal changes in the parenchyma of the affected testis compared to the unaffected side [12]. In cases of purulent lymphadenitis, ultrasound in the early stages of infection shows enlarged lymph nodes and preserved blood flow to the hilum, while that in the later stages of infection shows multiloculated fluid collection due to abscess formation accompanied by adjacent tissue edema [13]. The phase of infection is usually more advanced in cases requiring surgical incision compared to cases not requiring it [13]. Therefore, abscess formation may indicate an advanced phase of infection. Aggressive manual reduction under misdiagnosis could result in edematous changes in the gonads or adjacent tissue, and it may cause difficulty in sonographic diagnosis. In cases of lymphadenitis, manual reduction can cause a rupture, and differentiating between abscess formation and rupture is difficult. To prevent these difficulties in sonographic diagnosis after manual reduction for mimicker diseases, point-of-care ultrasound before manual reduction is important. For experienced physicians or surgeons, physical examination may be sufficient to diagnose incarcerated inguinal hernia; however, in cases with an atypical clinical presentation of incarcerated inguinal hernia, point-of-care ultrasound can provide useful information for the correct diagnosis.

## Data Availability

The data used to support the findings of this study are included within the article.
